# Advances in Functional Genomics for Exploring Abiotic Stress Tolerance Mechanisms in Cereals

**DOI:** 10.3390/plants14162459

**Published:** 2025-08-08

**Authors:** Tatenda Goche, Peter Mavindidze, Tinashe Zenda

**Affiliations:** 1 Crop Science Department, Faculty of Agriculture and Environmental Sciences, Bindura University of Science Education, Bindura P.O. Box 1020, Zimbabwe; 2 Department of Biosciences, Durham University, Durham DH1 3LE, UK; peter.mavindidze@durham.ac.uk; 3 Crop Science Department, Faculty of Plant and Animal Sciences and Technology, Marondera University of Agricultural Sciences and Technology, Marondera P.O. Box 35, Zimbabwe

**Keywords:** cereal crops, functional genomics, abiotic stress tolerance, third-generation sequencing, transcriptomics, genome-editing techniques, metagenomics

## Abstract

Climate change, population growth and the increasing demand for food and nutritional security necessitate the development of climate-resilient cereal crops. This requires first gaining mechanistic insights into the molecular mechanisms underpinning plant abiotic and biotic stress tolerance. Although this is challenging, recent conceptual and technological advances in functional genomics, coupled with computational biology, high-throughput plant phenotyping and artificial intelligence, are now aiding our uncovering of the molecular mechanisms underlying plant stress tolerance. Integrating other innovative approaches such as genome editing, modern plant breeding and synthetic biology facilitates the development of climate-smart cereal crops. Here, we discuss major recent advances in plant functional genomic approaches and techniques such as third-generation sequencing, transcriptomics, pangenomes, genome-wide association studies and epigenomics, which have advanced our understanding of the molecular basis of stress tolerance and development of stress-resilient cereals. Further, we highlight how these genomics approaches are successfully integrated into new plant breeding methods for effective development of stress-tolerant crops. Overall, harnessing these advances and improved knowledge of crop stress tolerance could accelerate development of climate-resilient cereals for global food and nutrition security.

## 1. Introduction

Crop plants often endure serious abiotic (drought, heat, salinity, anoxia, nutritional imbalances, etc.) and biotic (pathogenic, pest, etc.) stresses, which hinder their growth and development [1,2,3], consequently reducing crop productivity, quality and quantity [4,5]. The rapidly changing global climate renders these stresses increasingly important by escalating their incidence and severity [6,7,8]. At the same time, the global human population has increased to approximately 8 billion people [9], amplifying global food demand [1,10,11], against the backdrop of a shrinking natural agricultural resource base [12]. Consequently, agriculture faces a mammoth task to sufficiently and nutritiously feed the growing population, as scientists grapple with a climate adaptation–resource conservation–food security dichotomy [6,13]. Therefore, adaptation of cereals to climate change is critical in ensuring future food and nutrition security [14].

**Figure 1:**
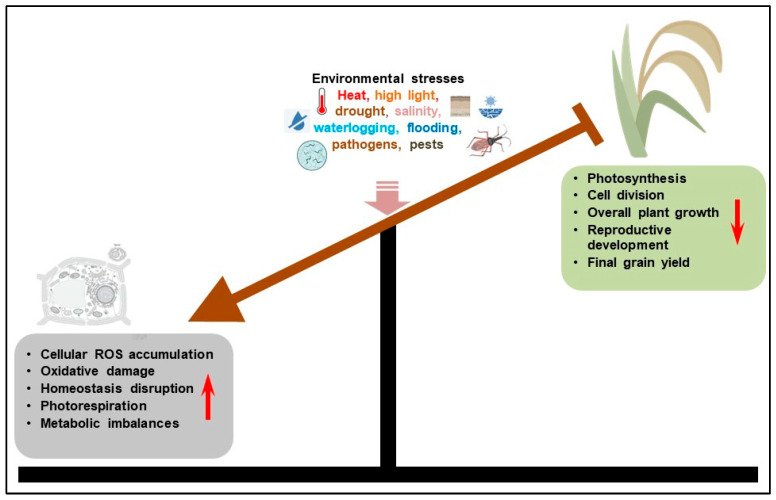
The impact of environmental stress on cereal crop plants. Environmental stresses repress plant physiological processes, overall growth and productivity, by increasing cellular reactive oxygen species accumulation, oxidative stress damage, homeostasis disruption and metabolic burden. Note: red arrows signify direction of change in parameters. Brown arrow and blunt ends signify promotive effect and repression, respectively.

Cereal crops such as wheat (*Triticum aestivum*), rice (*Oryza sativa*), maize (*Zea mays*), barley (*Hordeum vulgarie*) and sorghum (*Sorghum bicolor*) constitute a major portion of global human diets, with over 40% of calories directly derived from their grains, and another percentage from the use of cereals as animal feed and raw materials in beverages manufacture [15,16]. Consequently, the effects of climate change-exacerbated abiotic and biotic stresses on cereal crops pose huge global food and nutrition security challenges [16,17,18]. For instance, complex drought patterns have been shown to robustly explain global yield loss for major crops, with wheat, soybean (*Glycine max*) and maize yields across southern and eastern Europe, the Americas and sub-Saharan Africa exhibiting high and multi-crop susceptibility to more complex droughts than previously assessed [19]. Current yield loss estimates for soybean, wheat and maize for mid-century are 16%, 7.7% and 8.3%, respectively [20]. Meanwhile, a meta-analysis has revealed that combined drought and heat stress significantly reduce crop yields by diminishing harvest index, shortening crop life cycles and altering seed number, size and composition, with their impact being more severe during the reproductive stage of plants [21]. Both abiotic and biotic stresses disrupt several cellular and whole-plant functions, disturb cellular homeostasis and hinder physiological and metabolic processes, which negatively impact overall plant growth and development [22,23,24] (Figure 1). Therefore, gaining mechanistic insights into the fundamental aspects of crop stress tolerance and developing climate-resilient cereals could aid us in adapting agriculture to climate change and achieving global food and nutrition security [3,4,25,26].

Meanwhile, plants have evolved several mechanisms to tolerate abiotic and biotic stresses, including stress signal transduction, osmotic adjustment, antioxidant systems activation, transcriptional factor activation, phytohormonal regulation, defence-response-related or stress-responsive genes activation and epigenetic regulation [27,28,29,30].

Moreover, facilitated by epigenetic mechanisms, plants develop stress memory within their system, allowing them to ‘remember’ the initial stress when it recurs and institute apt responses [31,32]. Exploring the genetic and molecular mechanisms underlying cereal crops’ responses and tolerance to different abiotic and biotic stresses facilitates cereal crop improvement for climate resilience through targeted breeding [33,34]. In this regard, omics-driven elucidation of mechanisms underlying crop plants tolerance to environmental stress has significantly streamlined the identification of key candidate genes, proteins, metabolites and/or metabolic pathways linked to specific quantitative traits that have been successfully analysed (for instance, through quantitative trait mapping), validated, manipulated and/or incorporated into crop variety improvement programs much “earlier” than would the conventional approaches [35].

In the past, limited genetic information and resources hindered efforts to uncover the molecular basis of complex traits and identify stress-responsive genes in major cereals [34]. Although technologies like short-read Illumina sequencing have played a critical role in advancing genome sequencing projects [36,37], their inherent limitations, such as large assembly gaps and inability to span long genomic regions, have resulted in fragmented and error-prone assemblies. These shortcomings have, in turn, affected the accuracy of downstream analyses in many plant genomes [38,39]. Fortunately, rapid advances in nanopore technologies for single-molecule and long-read sequencing have facilitated the closing of gaps, improved accuracy, read length and throughput, yielded haplotype-resolved genome assemblies and simplified parsing of differences between plant genomes [40,41,42]. Evidently, therefore, the latest advances in functional genomics have greatly expanded our capacity to explore the genetic and molecular basis of plant stress tolerance and engineer climate resilience in cereals [25,43,44]. For instance, advanced genome sequencing techniques have facilitated the decoding of whole genomes, trait discovery and mapping of genomic regions controlling those quantitative traits in several cereal species [44]. RNA-seq transcriptomics analysis has successfully revealed key drought-responsive genes in maize and wheat [45,46], isobaric tags for relative and absolute quantitation (iTRAQ) proteomics has revealed key waterlogging-responsive proteins in wheat [47] and liquid chromatography combined with mass spectrometry (LC-MS) metabolomics analysis has successfully revealed key low-temperature stress-responsive metabolites in rice [48], etc. (summarised herein in Table 1).

Meanwhile, the now-available high-quality reference genomes [49,50], pangenomes [51,52,53,54], gene model annotations, expression atlases, single nucleotide polymorphisms (SNPs), genome-wide association studies (GWAS), gene networks, sequenced mutation populations, etc., constitute a repertoire of genetic and genomic resources underpinning functional genomics analysis of stress tolerance and breeding of crops [18,34,55]. These are supported by improved experimental and transformation protocols [56,57,58,59], high-throughput plant phenotyping platforms [60,61], multi-omics and single-cell omics [35,62] data analytics [63] and machine learning (ML) [64,65] approaches. Integrating these innovations with technological advances in biotechnology [66,67], genome editing [68,69,70] and modern breeding [18,71,72] approaches aids the exploration of stress tolerance and engineering of climate-resilience in cereals.

In this review, we discuss the various genomic approaches and tools that are driving the exploration of abiotic stress tolerance mechanisms in cereal crops and facilitating the development of climate-resilient varieties. We begin by briefly outlining the key mechanisms underlying plant responses to abiotic stresses, followed by an overview of genomics-based strategies used to investigate these mechanisms. Finally, we highlight how functional genomics is being integrated with modern plant breeding techniques to accelerate the development of stress-tolerant cereal crops.

**Table 1:** Selected comparative omics studies revealing abiotic stress-responsive genes/proteins/metabolites and tolerance mechanisms in major cereal crop species.

Abiotic Stress	Crop Species	Genotypes	Environmental Condition/Context	Outcome/Key Findings	Reference
**Transcriptomics**					
Drought and salinity	Wheat	Drought/salt tolerant JM22 and salt sensitive YM20	Genome-wide transcriptome analysis of wheat cultivars’ response to drought (10% soil moisture) and salinity (100 mM NaCl) stresses.	10 DEGs, annotated to cellular process, metabolic process, osmotic regulation, and MAPK signalling pathway, were co-identified as drought and salinity tolerance-associated DEGs contributing to better stress tolerance in JM22.	[45]
Drought	Maize	Drought-tolerant CML69 and susceptible LX9801 inbred lines	Comparative transcriptomic and physiological analyses of maize seedling leaf tissues response to 3–5 days of drought treatment.	Among other key results, the tolerant line showed significantly higher leaf RWC, and lower electrolyte leakage and MDA levels than the susceptible line, which possibly contributed to its better drought tolerance.	[73]
Waterlogging	Maize	Tropical line Suwan-2 and temperate line Cim-3	Comparative physiological and transcriptomic analysis of maize inbred line tolerance to waterlogging stress.	Crucial waterlogging-responsive DEGs in Suwan-2 were related to TF modulation, cellular redox homeostasis maintenance, and plant hormone biosynthesis regulation.	[74]
Salinity	Rice	Salt-tolerant HH11 and salt-sensitive IR29 cultivars	Transcriptome analysis of rice response to 200 mM NaCl salt for 0 h, 6 h, 24 h and 48 h at the 3 leaf stage.	HH11 showed more favourable antioxidant and osmotic adjustments than IR29 upon salt stress exposure, thus, better salt tolerance.	[75]
Heat stress (HS)	Wheat	Heat-tolerant genotype WH-730	Transcriptomic analysis of a heat-tolerant wheat genotype response to control and heat treatment conditions.	5610 heat-responsive DEGs were identified, and participate in HS response pathways, e.g., HSPs, antioxidant defence, and metabolic adjustments. Among them, peroxidase was dominant, enhancing HS tolerance, possibly via regulation of lignin biosynthesis.	[76]
Cold	Rice	Cold-tolerant cultivar Huaidao5 and Cold-sensitive cultivar Huaidao9	Differential expression and co-expression network analyses of rice panicle and flag leaf transcriptomes under reproductive-stage cold stress.	Huaidao5 showed better panicle tolerance to cold stress due to higher expression levels of cold-responsive genes in related pathways, e.g., MAPK signalling pathway, glutathione metabolism, plant hormone signal transduction, etc.	[77]
Cadmium	Rice	ZZ143 (low grain Cd) and YX409 (high grain Cd)	Genotypes subjected to 100 μmol/L Cd stress for 10 days.	ZZ143 showed higher root Cd tolerance than the susceptible genotype, possibly due to its greater root sulphur assimilation, and higher number of Cd-responsive DEGs and pathways, e.g., secondary metabolites biosynthesis, MAPK signalling, etc.	[78]
Low nitrogen	Sorghum	N-efficient (398B) and the N-inefficient (CS3541) inbred lines	Comparative phenotypic and transcriptome analysis of sorghum genotypes under low N hydroponic and field conditions	398B exhibited superior low N tolerance than CS3541 under both field and hydroponic conditions, due to its higher photosynthetic performance and sustenance of N metabolism-related enzyme activities.	[79]
**Proteomics**					
Drought	Wheat	Tolerant BW35695 and drought-sensitive BW4074	Physiological, biochemical, and iTRAQ leaf proteome analyses of wheat responses to drought.	Tolerant variety showed greater osmotic adjustment, antioxidant capacity, and high upregulation of protein synthesis-related proteins, contributing to better stress tolerance.	[80]
Drought	Maize	Drought-tolerant YE8112 and drought-sensitive MO17	Physiological and iTRAQ leaf proteome analyses of maize responses to drought.	A total of 721 DAPs were identified. Most DAPs in YE8112 were associated with photosynthesis antenna proteins pathway, and YE8112 had better tolerance due to its activation of photosynthesis proteins related to balancing light capture and utilisation.	[81]
Heat	Rice	Heat-tolerant variety 9311 and sensitive variety Guangluai4 (GLA4)	Phosphoproteomic analysis of high temp (30–38 °C for 1 to 9 days)-induced changes in indica rice developing grains.	A total of 9994 phosphosites from 3216 phosphoproteins were identified in all endosperm samples. Several HS-induced consensus phosphorylation motifs were identified, and revealed a core set of HS-responsive protein kinases, splicing factors, and regulatory factors, especially those involved in starch metabolism.	[82]
DS and elevated temp (ET)	Barley	7 spring barley RILs (hybrids of European and Syrian accessions)	LC-MS based proteomic analysis of barley flag leaf response to drought and ET (20/30 °C night/day).	Several protein accumulation changes under DS, ET and combined stresses were identified, including for photosynthetic apparatus-related proteins. Dehydrins were found among universally stress-responsive proteins.	[83]
Waterlogging	Wheat	Tolerant XM 55 and sensitive genotypes YM 158	iTRAQ proteomic analysis of wheat responses to waterlogging stress.	Of the 7710 DAPs identified, 16 were distinct between the 2 cultivars under stress; 11 DAPs were upregulated and 5 were downregulated. 9 DAPs, including DEAD-box ATP-dependent RNA helicase 3, responded to waterlogging with non-cultivar specificity.	[47]
Salinity	Pearl millet (*Pennisetum glaucum*)	Tolerant (Tol) and sensitive (Sen) accessions	2DE-based whole proteome analysis of pearl millet response to 150 mm NaCl treatment	295 and 315 protein spots were identified in tolerant and sensitive accessions, respectively. Salinity tolerance of the tolerant accession was attributed to its higher upregulation of stress-responsive proteins.	[84]
Salinity	Wheat	Kharchia-65 salt-tolerant) and PBW-373 (salt-sensitive)	LC–MS/MS based proteomic analysis of wheat responses to 0 and 300 mM NaCl treatment for 48 h.	21,863 proteins and 5133 protein groups were identified. There was higher upregulation of stress-responsive proteins, e.g., auxin-responsive, peroxidase, etc., in tolerant genotype and comparative downregulation in susceptible genotype.	[85]
Low temperature (LT)	Maize	LT-tolerant Gurez local and LT-sensitive GM6	2D-PAGE based proteomic analysis of maize leaf responses to low temp (6 °C) exposure for 12 h at 3-leaf stage.	19 and 10 proteins were identified in Gurez local and GM6, respectively, including 3 novel abiotic stress- and LT-responsive proteins (e.g., nodulin-like protein) identified from Gurez local.	[86]
Aluminium (Al)	Barley	Al-sensitive barley cultivar ZU9	TMT-based quantitative proteomic analysis of barley response to aluminium stress under phosphorus-*Piriformospora indica* interaction	DEPs were mostly enriched in the phenylpropanoid biosynthesis pathway, among which peroxidases were prominent. *P. indica* in combination with P helped barley plants to endure Al-induced stress by modulating antioxidative defence system.	[87]
Low inorganic phosphorus (Pi)	Wheat	Higher PUE genotype TM98 and a lower PUE genotype H4399	Label-free quantitative proteomic analysis of wheat leaf response to low Pi.	2110 high-confidence proteins were identified, among them 244 and 133 DAPs under Pi deficiency in H4399 and TM98, respectively. Abundance of energy metabolism-related proteins was decreased by Pi deficiency in H4399 shoots, but not in TM98.	[88]
Drought	Sorghum	Drought-sensitive S4 and S4-1, and drought-resistant T33 and T14	nano-LC-MS/MS-based leaf proteome analysis of sorghum response to drought.	A total of 3927 proteins were quantified, with 46, 36, 35, and 102 DAPs identified in S4, S4-1, T14, and T33 varieties, respectively. Tolerant genotypes showed enhanced TCA cycle and influenced aminoacyl-tRNA biosynthesis.	[89]
**Metabolomics**					
Low temp (LT)	Rice	Varieties 02428 (*japonica*) and YZX (*indica*)	LC–MS/MS-based metabolomics analysis of rice response to LT (15 °C for 4 days) at germination.	A total of 730 metabolites were detected by LC-MS/MS method. 7 key LT-responsive metabolites were identified, and these metabolites were observed to participate in biosynthesis of amino acids and phenylpropanoids, as well as metabolism of glutathione and inositol phosphate.	[48]
Low nitrogen	Wheat	Zheng Mai 366 and Ai Kang58, dominant species in Henan.	UPLC-QTOF-based analysis of wheat flag leaf response to low N stress.	Chemical analyses identified 11 secondary metabolites, considered biomarkers of low N stress. Most of these secondary metabolites were flavonoids and their related derivatives, such as iso-vitexin, iso-orientin, etc.	[90]
Low nitrogen	Sorghum	10 diverse entries (including inbreds and hybrids)	UPLC-MS/MS-based analysis of sorghum roots’ response to low N.	Roots from plants with N stress contained reduced phenylalanine, a precursor for salicylic acid, providing evidence for compromised metabolic capacity for defence response under low N conditions.	[91]
Drought	Barley	German variety Maresi and Syrian breeding line Cam/B1//CI08887/CI05761	Untargeted GC-MS based metabolomics profiling of barley leaf and root responses to drought.	Compatible solutes and osmolytes were the major group of compounds accumulated under drought, and revealed changes in accumulation of some metabolites, e.g., proline and other amino acids, CHOs or carboxylic acids were considered a basic plant strategy for acquiring drought stress tolerance.	[92]
Drought	Wheat	Drought-tolerant T13 and drought-susceptible T2	Integrated transcriptome and metabolomics analyses of wheat responses to drought.	Flavonoids and phenolic acids metabolism were associated with wheat seedlings’ drought tolerance, with their biosynthesis-related DEMs and genes possibly being key factors underlining the difference in drought tolerance.	[93]
Low phosphorus (LP)	Wheat	G28 (LP-tolerant) and L143 (LP-sensitive) varieties	Metabolomics and transcriptomics analysis of wheat response to 72 h of LP stress.	A total of 181 and 163 DAMs were detected in G28LP and L143LP under LP stress, respectively. Additionally, joint metabolomics and transcriptomic analysis revealed that wheat LP tolerance was closely related to 15 metabolites and 18 key genes in the sugar and amino acid metabolism pathways.	[94]
Salt and heat	Wheat	Warm-adapted Fahng60 and heat-sensitive Samerng2 cultivars	Physiological and metabolomics analysis of seedlings’ response to salt (150 mM NaCl) and HS (42 °C for 4 h) treatments.	Amino acids, sugars, and sugar derivatives were the major responsive metabolites in leaves under the stress. Additionally, in both genotypes, the ABC transporters, glucosinolate metabolism, aminoacyl-tRNA biosynthesis, etc., were the key overrepresented pathways under the stress combination.	[95]
Saline-alkaline	Rice	Saline–alkali-tolerant cultivar Tongxi926	Integrated transcriptome and metabolomics analysis of rice response to high saline–alkali stress (pH > 9.5).	9347 DEGs and 693 DAMs were identified. Among the DAMs, lipid and amino acid accumulation were greatly enhanced, and pathways related to ABC transporter, amino acid biosynthesis, glutathione metabolism, TCA cycle, etc., were significantly enriched.	[96]
Drought	Maize	Drought-tolerant line si287 and a drought-sensitive line X178	Transcriptomic and metabolomics analysis of maize response to a 7-day drought at the 3-leaf stage	DEGs and DEMs were significantly enriched in flavonoid biosynthesis, starch and sucrose metabolism, and amino acids biosynthesis-related pathways. Joint analysis identified proline, tryptophan and phenylalanine as key stress-responsive amino acids.	[97]
Salinity	Barley	GN2 (salt-tolerant) and GN18 (salt-sensitive)	Proteomic and metabolomics analysis of barley response to salt stress at germination stage.	Besides the stress-responsive DAPs, a total of 187 salt-regulated metabolites were identified, which were mainly related to ABC transporters, amino acid metabolism, CHO metabolism and lipid metabolism.	[98]

Note: DEGs, differentially expressed genes; DAPs, differentially abundant proteins; DEPs, differentially expressed proteins; DEMs, differentially expressed metabolites; DAMs, differentially accumulated metabolites; PUE, phosphorus utilization efficiency; HSPs, heat shock proteins; CHO, carbohydrates; Cd, cadmium; iTRAQ, isobaric tags for relative and absolute quantitation; LC-MS/MS, liquid chromatography combined with mass spectrometry; nano-LC-MS/MS, nano-scale liquid chromatography mass spectrometry; 2D-PAGE, two dimensional gel electrophoresis; LFQ-based, label-free quantification based; UPLC-QTOF, ultra-performance liquid chromatography-quadrupole time-of-flight mass spectrometry.

## 2. Overview of Abiotic Stress Tolerance Mechanisms in Cereals

Creating stress-tolerant crops requires first gaining a mechanistic understanding of the plant biological processes and fundamental mechanisms underlying responses to those different stresses [1,99]. Cereals have evolved to thrive in environments that routinely expose them to a variety of stressors, such as high temperatures, drought, salt, mineral toxicity and water scarcity. To cope with these pressures, cereals have developed a variety of stress tolerance mechanisms. These include the accumulation of compatible solutes such as proline, betaine and sugars to maintain cell turgor and prevent water loss [100,101,102,103,104]. In addition, cereals can cope with drought stress by reducing transpiration rates and increasing water-use efficiency [105,106]. They can also produce deeper roots to access deeper soil water reserves [107,108]. Furthermore, cereals possess the capacity to withstand high temperatures due to their ability to release heat shock proteins, which protect proteins from denaturation and oxidative damage [109]. To withstand cold stress, some cereals produce antifreeze proteins, which inhibit ice from forming and shield membranes from damage [110,111]. Cereals also possess an inherent ability to tolerate high salt concentrations by excluding salt from the roots and accumulating it in the vacuoles, as well as balancing the expression of reactive oxygen species (ROS)-scavenging genes [104,112,113,114,115,116,117].

Understanding root development and architecture holds the promise of exploiting and manipulating root traits to boost food plant output and optimise agricultural land usage. Cereals alter their root architecture to increase nutrient uptake under stress. The plants accomplish this via the production of enzymes that aid in nutrient mobilisation and assimilation. Siddiqui et al. [118] and Halder et al. [119] detail the genomics of root system variation and root system architecture under abiotic stress, respectively.

Under field conditions, different environmental stresses usually occur concurrently. The simultaneous occurrence of several environmental stressors and biotic variables triggers complex plant responses due to crosstalk mechanisms. Combined stresses are usually more damaging to plant systems in comparison to individual stress [120]. Combined heat and drought stress on winter wheat inflicted more damage compared to individual stress and the recovery rates of the physiological traits affected by the combined stress [121]. Another research team has also highlighted the complexity of combined stress on sorghum [122]. Understanding these mechanisms is essential for creating stress-tolerant cereal cultivars that can ensure food security in the face of climate change. Rigorous approaches are required in the development of multi-stress-tolerant cereal crops. Schimt et al. [123] identified novel alleles for combined heat and drought stress in wheat, whilst Yang et al. [124] showed that the maize primary root response to combined heat and drought stress is regulated via the salicylic acid pathway. Further combined stress studies have been performed in maize [124,125], wheat [126,127,128], rice [129,130], sorghum [131] and barley [132].

## 3. Recent Advances in Crop Functional Genomics

In this section, we briefly discuss various genomic approaches underpinning the exploration of stress tolerance mechanisms in crop plants. These include genome sequencing and annotation, transcriptomics, proteomics and metabolomics, among others.

### 3.1. Third Generation Sequencing, Long Reads and Pangenomes

The evolvement of genomics technologies over the past decade has revolutionised genome sequencing, annotation and analysis, which has advanced our understanding of plant genetic architecture of environmental adaptation [44,50,133]. Cost-effective high-throughput next-generation sequencing (NGS) approaches (Illumina/Solexa, Ion Torrent PGM, etc.) have enabled whole-genome sequencing of most crop species [44,50,134,135,136]; however, NGS approaches produced fragmented assemblies due to short-read length, which have low accuracy, hence are not able to detect low-abundance mutations and SNPs [25]. Fortunately, the current third-generation sequencing (TGS) technologies, especially Oxford Nanopore and Pacific Bioscience platforms [137,138], anchored on long reads [49,133], have facilitated the assemblage of high-quality chromosomal-scale plant genomes [25] and pangenomes for most major crops, some minor species and crop wild relatives (CWRs) [35,139,140]. This has offered us a holistic 360-degree view of the plant genomes, viz., the structure, organisation and regulation [41], as well as facilitating the mining of novel agronomic and climate-responsive alleles from CWRs through de novo domestication [25,141,142,143,144].

Despite short-read sequencing being cost-effective, accurate and amenable to diverse analysis tools and pipelines, it cannot span lengthy nucleic acid polymers and complicates reconstruction and counting of the original molecules [40]. On the other hand, long-read sequencing (LRS) allows for gap closing and improved accuracy. TGS approaches now offer possibilities to resolve transposon repeats through generation of long reads that span over transposon regions, including unknown locations, thereby facilitating the production of high-quality assemblies for complex genomes [10,145]. These LRS-based methods combine nanopore and single-molecule real-time sequencing to yield long reads (of ~60–200 Kb length), which can span complex and repetitive regions [10,133,146]. They are usually coupled to optical mapping and confirmation capture to generate draft genomes of unmatched contiguity [25,147]. Thus, long reads can significantly enhance de novo assembly, mapping certainty, transcript isoform detection and structural variant calling. Furthermore, LRS of native DNA and/or RNA molecules removes amplification bias while preserving base modifications [40,148]. Taken together, the era of LRS approaches is offering a window for de novo assembling of genomes, resolving sequence assembly ambiguities and gap filling. Moreover, this has facilitated comparisons between related species and identification of subtle genetic variations that may be key in improving elite crops [44].

Molecular markers, especially genome-scale SNPs, now permit quick dissection of genome-wide variations and key agronomic and climate-adaptive traits, from both elite and CWRs, which is essential for traits introgression, marker-assisted selection (MAS), marker-assisted breeding (MAB) and other genomic applications [43,149,150]. The greater abundance of SNPs permits greater coverage of loci across the genome [151]. Moreover, SNPs are amenable to high-throughput (HTP) and automated profiling, thereby facilitating rapid and HTP high-density SNP-marker-based genotyping [152,153]. Thus, SNPs are now widely deployed (more than other genetic markers such as single-sequence repeats) in marker-assisted breeding, GWAS, genomic selection (GS) and map-based cloning [151,154,155].

Feasibly, high-resolution GWAS has been conveniently applied to identify key genetic factors (QTLs, genes’ genomic regions and haplotypes) governing several key agronomic, climate-adaptive and nutritional traits [35,149,156,157,158,159,160,161]. For instance, recent advancements in functional genomics approaches and resources have unprecedentedly facilitated functional characterisation of genes in crops with large genomes, such as polyploidy wheat (AABBDD), which has aided crop improvement [34]. Integrating these novel genetic and genomic resources (including high-quality draft genomes, pangenomes, gene model annotations, expression atlases, gene networks, sequence indexed mutant population, structured natural population, omics databases, etc. [18,34,149,162]) offers rapid and comprehensive methods for gene functional analysis, which enhances our understanding of the molecular mechanisms underpinning crop climate adaptation and accelerates the designing of climate-resilient crops [18].

### 3.2. Transcriptomics

The comprehensive examination of all expressed RNA transcripts a given organism has produced is known as transcriptomics. This covers not only the conceptual changes in the genome but also the mechanism through which the information stored in the genome is utilised by the cell, as well as the flow of biological information from the genome to the cell. Advances in transcriptomics include RNA sequencing (RNA-seq), gene co-expression network analysis, alternative splicing analysis, long non-coding RNA (lncRNA) analysis and functional annotation and pathway analysis. RNA-seq is a technique that uses NGS to investigate the quantity and sequences of RNA in a sample. It examines the transcriptome to determine which genes encoded in DNA are activated or suppressed and to what degree [163]. RNA-Seq, as the technology of choice for whole-transcriptome studies, has been successfully employed in cereal crop stress response studies, offering accurate gene expression profiling analysis and revealing key stress-responsive genes, metabolic pathways and stress tolerance mechanisms [164,165,166,167] (Table 1; Supplementary Table S1). Its applications are of great value in cereal crop gene functional validation. For example, RNA-seq and qRT-PCR were utilised to confirm that the overexpression of the heat shock factor gene (*TaHsfA6bT*) from wheat provides thermotolerance in barley [168].

Gene co-expression network analysis is a computational tool for identifying gene modules that are co-regulated under stress situations. This technique can assist in the identification of essential regulatory mechanisms involved in cereal stress tolerance, for example, drought stress tolerance in maize [169]. Critical modules and genes for drought tolerance in wheat have been identified using this technique [170]. In addition, Chopra et al. [171] identified complex gene interactions for cold and heat stresses in sorghum after evaluating their co-expression networks. Furthermore, tandem maize paralogues were shown to be consistently co-expressed under cadmium (cd) stress in a study that elucidated the genetic basis of plant response to cd stress. Alternative splicing involves the production of several mRNA isoforms from a single gene, and alternative splicing variation can be exploited in crop improvement programs for stress tolerance [172]. Lin et al. [173] performed alternative splicing of *ZmrbohB* cDNA to obtain two transcript isoforms in maize. Transcriptomics can be used to investigate alternative splicing events in response to stress [174]. This method can aid in the discovery of new transcripts and isoforms implicated in stress-response pathways. Mastrangelo et al. [175] comprehensively detailed transcriptomic plasticity through alternative splicing.

Long non-coding RNAs (lncRNAs) are a type of non-coding RNA that plays a role in gene expression regulation [176]. They have been defined as transcripts longer than 200 nucleotides with limited phylogenetic conservation, expressed at low levels and characterised by tissue/organ-specific expression profiles [177]. These transcripts have been characterised and shown to be responsive to drought, salt, cold, boron and heat stress in studies on maize [178,179,180,181,182,183]. Novel lncRNAs engaged in stress-response pathways have been identified concurrently. Further research has been carried out in sorghum, wheat, barley and the closely related *Brachypodium distachyon* [184]. Overall, improvements in transcriptomics have significantly increased our understanding of the molecular pathways underlying cereal stress tolerance. This knowledge can be utilised to create stress-tolerant cultivars that are better suited to changing environmental conditions.

### 3.3. Proteomics

The proteome offers a unique avenue for the study of intricate biological functions involving vast numbers and networks of proteins. Protein identification makes use of a variety of technologies. These are categorised into gel-based and non-gel-based techniques, coupled with mass spectrometry and bioinformatics for putative functional annotation. Recent developments in mass spectrometry (MS)-based tools and methodologies have led to a rise in the use of a variety of unique proteomics approaches on cereals. Traditionally, two-dimensional polyacrylamide gel electrophoresis (2-D PAGE) or two-dimensional electrophoresis (2-DE) [185] has been used as a standard procedure for proteomics research. However, gel-based technologies are labour-intensive and time-consuming, making them unsuitable for processing large quantities of samples. MS-based methods such as the iTRAQ are the most widely used technology for identifying, characterising and quantifying proteins and their proteoforms, mainly because they offer high throughput and applicability to large sample numbers [186]. Genomics and transcriptomics are concerned with genes present in a genome and their expression, whereas proteomics is concerned with the qualitative and quantitative components of expressed proteins, as well as how the expressed levels fluctuate under different environmental situations. Aside from protein abundance, the existence of protein modifications can help in understanding biological processes or chemical adjustments such as signalling and food processing, respectively [187,188].

Proteomics has emerged as a powerful tool for exploring stress tolerance in cereals as it allows researchers to identify and quantify differentially expressed proteins in response to different stresses (Table 1; Supplementary Table S2). The availability of cereal crop complete genome sequences is a significant step forward in proteomics studies since it allows for the identification of genes linked with essential agronomic features. Advances in proteomics for exploring stress tolerance in cereals include the identification of stress-responsive proteins [89,107,189], the mapping of protein–protein interactions [190,191], the quantification of protein expression levels [89,192] and the development of stress-tolerant cultivars [193,194,195]. Some of the milestones achieved in cereals include the identification of heat shock proteins, dehydrins and late embryogenesis abundant (LEA) proteins that are upregulated in response to various stresses in maize and sorghum [107,189], protein complexes involved in the response to salt stress in rice [190] and changes in the expression levels of enzymes involved in the synthesis of compatible solutes in response to salt stress in barley [192] (Table 1; Supplementary Table S2). In addition, proteins involved in stress response, with potential to be targeted for crop genetic improvement, have been identified in most cereals, as recently reviewed [196].

### 3.4. Metabolomics

Metabolomics technologies speed up crop development research through the discovery of different metabolic pathways for abiotic/biotic stress tolerance. Metabolites have been regarded as “the ultimate response of biological systems to genetic or environmental changes” since they constitute one quantifiable endpoint of all cellular regulatory functions [197]. Modern metabolomics methods, such as improved mass spectrometry (MS), nuclear magnetic resonance (NMR) and cutting-edge imaging technologies like nanoscale-secondary ion mass spectrometry (NanoSIMS), have created new opportunities for in-depth study of the cereal metabolome. The full spectrum of techniques that can be used to study the plant metabolome has been extensively reviewed [198].

Metabolomic analyses can be classified as directed/targeted or undirected/non-targeted [199]. Directed metabolomics entails focused analyses that aim to identify and quantify as many metabolites within a given chemical group as possible, whilst the goal of undirected metabolomics is to detect as many metabolite groups as possible in order to create patterns or fingerprints, with or without identifying the identified molecules or compounds. Stress-related amino acids have been identified in tropical sorghum through non-targeted metabolomics [200]. Through directed metabolomics, Sheflin et al. [91] identified and quantified phenylalanine, a precursor for salicylic acid, providing evidence for compromised metabolic capacity for defence response under low-nitrogen conditions.

In contrast to the transcriptome and proteome, the metabolome is not necessarily linked to the plant genome [201]. It is, however, closely linked to the phenotype [202], providing a perfect platform to probe stress-responsive metabolites and stress-induced regulatory pathways that govern abiotic stress tolerance (Table 1). However, there is a gap between metabolite expression and the sophisticated stress tolerance process for identifying desired metabolites that operate effectively under stress conditions. Filling this gap could aid in the development of an effective breeding plan and facilitate the gathering of data for the identification of cultivars that perform well under abiotic stress conditions. In an effort to address this, Zhang et al. [203] successfully used a multi-omics approach to reveal the roles and mechanisms of two hub genes, *Bx12* and *ZmGLK44*, in regulating maize metabolite biosynthesis and drought tolerance.

## 4. Genome Editing Technologies

Over the last decade, genome editing has emerged as a novel approach that allows researchers to directly control literally every gene in a wide variety of cell types and organisms. Genome-editing technologies are tools that enable the modification of individual genes to understand their roles [204]. Technologies developed for genome editing include the Clustered Regularly Interspaced Short Palindromic Repeats-CRISPR-associated protein 9 (CRISPR-Cas9), CRISPR-Prevotella and Francisella 1 (CRISPR-Cpf1), Transcription Activator-Like Effector Nucleases (TALENs) and Zinc Finger Nucleases (ZFNs). The CRISPR-Cas9 enables precise and efficient gene editing by utilising a guide RNA (gRNA) to target a specific DNA sequence and the Cas9 enzyme to introduce changes, such as gene knockouts or precise gene modifications. CRISPR-Cas9 has been widely applied in cereal crop research to better understand the function of certain genes implicated in stress response, including drought in maize [205], heat in several species [206], chilling stress in rice [207] and salinity in rice and wheat [208]. This is due to its simplicity, accessibility, adaptability, flexibility and wide applicability. CRISPR-Cpf1 is similar to CRISPR-Cas9, except instead of Cas9, the Cpf1 (Cas12a) enzyme is used. CRISPR-Cpf1, like CRISPR-Cas9, can be utilised for precise gene editing, such as gene knockouts and alterations. It has various advantages over Cas9, such as a reduced gRNA size and different target site preferences.

ZFNs and TALENs are chimeric nucleases made up of sequence-specific DNA-binding modules coupled to a non-specific DNA cleavage domain [209,210]. These nucleases enable a wide range of genetic alterations by causing DNA double-strand breaks, which drive error-prone non-homologous end joining or homology-directed repair at specified genomic loci [211]. The programmability of the DNA-binding domains that are derived from zinc-finger and transcription activator-like effector proteins has previously established these technologies as the most preferred in genetic engineering. CRISPR-Cas9, ZFNs and TALENs, however, faced challenges that CRISPR-Cpf1/Cas12a managed to fill [212]. These include re-engineering at the same target DNA region. This was because of the loss of the target site after the initial engineering with CRISPR-Cas9, ZFNs or TALENs. Cpf1 (Cas12a) functions as both an endoribonuclease and an endodeoxyribonuclease, cleaving target sequences and generating double-stranded breaks. Cpf1 also enables multiplexed genome editing because a single crRNA array transcript can target several loci in the genome. Although many successes were reported on model cereal crops such as rice [213,214,215,216], initial CRISPR-Cpf1 research in other cereals required optimisation to improve its applicability [217].

Overall, these genome editing technologies can be used to explore the function of genes involved in cereal crop stress responses. The involvement of genes of interest in stress tolerance systems can be ascertained, and plants with greater stress resistance can be designed by precise alterations. However, it is critical to evaluate the ethical and regulatory elements of genome editing technologies in order to ensure their responsible and safe use.

## 5. Epigenomics

Environmental stresses induce epigenetic changes such as cytosine DNA methylation and histone post-translational modifications, which alter the chromatin structure and spatiotemporal gene expression. Stable epigenetic alterations are inheritable across filial generations, and this facilitates plant adaptation to environmental change [218]. Thus, epigenomics, which involves the study of chromatin states, chromatin alterations, their impact on gene regulation and aspects of epigenetic inheritance [219,220], has emerged as a new avenue for exploring molecular mechanisms of tolerance and improving crop adaptation to environmental stresses [221,222,223,224]. Epigenomics leverages chromatin information to better annotate and decode plant genomes and offers complementary strategies to identify and select heritable epialleles that govern crop traits independent of underlying genotype [220]. Considering that epigenetic mechanisms play crucial roles in other biological processes and stress responses [225,226], gaining mechanistic insights into the stress-induced epigenetic changes in plants helps us better understand the molecular adaptation of plants to environmental stresses [218,219]. Additionally, elucidation of the epigenetics-mediated cryptic molecular switches that drive the capacity of plants to memorise stress and reproduce stress-resistant progenies will help us in breeding climate-resilient crop species [221]. Leveraging recent advances in genome analysis tools, several epigenetic resources, including epialleles, epigenetic quantitative trait loci (epiQTLs), epigenome-wide association (epiGWAS), etc., can be utilised in epibreeding for improved stress tolerance in crops [72,218,227,228].

Meanwhile, plant microRNAs (miRNAs) [229,230] and long non-coding RNAs (lncRNAs) [231] have emerged as key post-transcriptional and translational regulators of gene-expression related to growth, development and stress response modulation [44,232]. To date, several stress-responsive miRNAs have been identified in different crops (including miR156, miR159, miR168, miR398, etc.) and their key roles in epigenetic regulation of stress responses established [233,234,235,236]. Meanwhile, miRNA-driven RNA-interference (RNAi) is rapidly becoming the preferred technology for improving crop traits and plasticity to environmental stress [237]. Thus, epigenetics can be integrated into the analyses of non-coding RNA, cis-regulatory elements and other non-genic variations controlling plant stress adaptation, thereby facilitating epigenetics-assisted breeding of crops [35,44,224,238]. Furthermore, supported by new epigenome editing tools, epigenetic engineering has already been initiated [218]. For instance, target-specific epigenetic engineering has been attempted by exogenous RNAi, facilitated by VIGS (virus-induced gene silencing) and grafting [225]. Going forward, new innovative techniques such as CRISPR-Cas9 and synthetic biology are anticipated to anchor epigenetic engineering of plant genomes, making epibreeding of crops for stress tolerance possible [225,239]. Further, induced epigenetic modifications can be a source of phenotypic variations in natural populations that can be passed on to multiple filial generations [240]. In light of the decreased genetic variation in conventional crop breeding due to selection pressure and climate change [67,225], epigenetic modifications may contribute to crop breeding by providing epigenome diversity, useful markers and eustressors for use in epibreeding of climate-resilient crops [222,223,225,226].

## 6. Integrating Novel Breeding Methods for Quick Trait Fixation and Optimization

Despite being characterised with comparably longer periods (~7–10 years), contemporary plant breeding approaches significantly anchored the identification and introgression of several key agronomic and stress-responsive traits/genes in several crops in the past [33,66,158,241,242,243,244]. However, the fast-changing climate dictates that rapid breeding cycles should be achieved, and, thus, calls for new breeding innovations [8,245]. Building on the recent advances in functional genomics, we can now innovate with several new breeding methods for improved selection and breeding efficiency, quick fixation of key agronomic and climate-adaptive traits and optimisation of plant performance [71,246]. Top among these novel approaches are genomic selection [247,248,249], speed breeding [250], haplotype-based breeding [251], fast-forward breeding [252] and smart breeding [253].

Genomic selection (GS) now offers unprecedented opportunities for improving selection accuracy, minimising phenotyping, reducing cycle time and increasing speed and genetic gains of the breeding programs [71,254,255,256]. GS can facilitate selection for complex or polygenic traits (governed by multiple additive effects genes) such as drought and/or heat stress tolerance [10], by means of the net genetic merit of an individual obtained by making use of several genome-wide markers [257]. Unlike MAS, which relies on the association between the trait of interest and other genes/traits [258], GS is practically viable even when the causal association is not known [254]. In GS, the individual effects of genome-wide HTP markers are estimated through prediction models [249]. Then, genomic-estimated breeding values (GEBVs) of these individuals are computed to select the elite lines; this does not require phenotyping [247,259]. Two populations are used, i.e., a training or reference population comprising a cohort of individuals with known genotypic and phenotypic information, and a testing or breeding population comprising candidate breeding lines with genotypic data only [247,254]. In the present climate change scenario, GS holds much possibility for improving the genetic gain of breeding programs and ensuring rapid development of climate-tolerant crops, including cereals [154,255,256].

NGS and TGS-based approaches and SNP arrays permit and expedite genotyping by sequencing (GBS) of large germplasm pools for GWAS and GS [153,155], as well as facilitating haplotype-based breeding [251,260]. These innovations enhance the capturing of species-wide genetic variation and increase the efficiency of breeding programs, as ML can be used to analyse genotypic data [261]. Meanwhile, speed breeding and GETs considerably accelerate the development of climate-resilient crops [7,143]. Speed breeding involves nurturing thousands to billions of plants under fully enclosed glasshouses and artificial light-controlled conditions [250] and considerably shortens the plant growth cycles, thereby fast-tracking crop improvement programs [262]. For instance, up to seven generation cycles per year can be attained in chickpea [263], which considerably saves breeding and variety release time by ~50%. Moreover, by shortening the breeding cycles, speed breeding can be used to investigate a range of physiological indices and optimise the transgenic pipeline, gene stacking, GS and genome editing for crop improvement [264].

**Figure 2:**
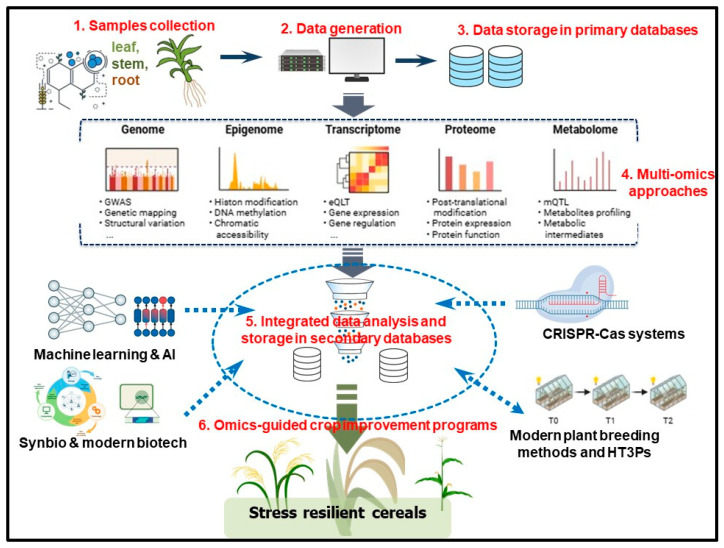
Integration of multi-omics approaches to modern plant breeding, biotechnology, machine learning, artificial intelligence (AI) and other technologies could considerably accelerate the identification and introgression of novel and key stress-responsive traits into elite crops, consequently fast-tracking the development of new stress-tolerant cereals. Note: HT3Ps, high-throughput plant phenotyping platforms; Synbio, synthetic biology; light blue arrows and circles signify integration.

Haplotype-based breeding (HBB) involves the identification of superior haplotypes and their deployment in breeding programs [265]. HBB circumvents the challenges faced by SNPs in predicting complex traits, increases the resolution of candidate genomic regions [251] and enhances exploitation of natural genetic variation within genetic populations, consequently improving breeding efficiency and precision [266]. Fast-forward breeding integrates TGS, high-throughput plant-phenotyping platforms (HT3Ps), systems biology, QTL mapping, GS and genomic prediction, machine learning (ML), artificial intelligence (AI) and other novel breeding approaches to significantly enhance the genetic base of breeding programs and accelerate genetic gains, thereby quickening the creation of stress-tolerant crop cultivars [252]. Smart breeding, on the other hand, is driven by big data (gathered spatiotemporally over multiple-environmental trial sites), AI, optimised prediction models and integrated genomic-enviromic prediction (iGEP) [253] and provides a platform for coupling multi-omics information to ML and AI for targeted designing of crop breeding pipelines [253]. Furthermore, HT3Ps, including novel sensors and high-resolution imagery, can quantify plant performance in specific environments with the generated phenomics data optimised for genetic gains [61,253,267]. Taken together, integrating novel plant breeding methods with functional genomics approaches considerably accelerates the identification and introgression of novel and key stress-responsive traits into elite crops, consequently fast-tracking the creation of new stress-tolerant crop cultivars [25,44,71,246,262] (Figure 2).

## 7. Metagenomics

A cocktail of mutualistic microorganisms (bacteria, fungi, etc.) inhabits plant surfaces, interacting with each other and with their plant hosts intimately [268]. These microbial assemblies constitute the dynamic plant microbiome and are critical for plants’ survival [269,270]. Plant microbiomes confer health and fitness benefits to their plant hosts, such as providing novel nutritional and biosynthetic capacities that invigorate plant growth and improve resistance to different abiotic and biotic stresses [270,271]. Recently, plant microbiomes have been reported to enhance plant capacities for disease resistance [272] and tolerance to abiotic stresses such as drought [273], heat [274] and salinity [29,271,274,275,276]. Furthermore, they influence plant metabolism and phytohormonal pathways aimed at instituting enhanced plant fitness and survival [270]. Thus, at the moment, plant microbiomes are a hot topic for research for improved plant stress tolerance and crop climate resilience [29,276,277,278,279].

A global analysis of the heterogeneity and exact functions of the plant microbiomes will provide new insights into the mechanisms of plant stress response and tolerance to biotic and abiotic stresses [270,280]. Moreover, beneficial interactions, such as nitrogen fixation, can be harnessed to enhance crop productivity and environmental functioning [281,282]. Despite the associated conceptual and technical bottlenecks, metagenomics is a powerful tool that can facilitate rapid analysis of microbial heterogeneity, thereby aiding us in deciphering the associations among microorganisms and their environment, as well as the overall functioning of the microbiome [275,280]. Buoyed by the recent conceptual and technological advancements in metagenomics and metatranscriptomics, computational biology [283], ML and HT3P platforms [272,284], as well as the availability of huge microbiome datasets generated from genome sequencing [285,286], identification and analysis of microbiomes is increasingly becoming feasible and meaningful. In particular, the recent progress in metabolomics methods and data analysis tools is anticipated to play a significant role in this regard [280,287].

Among other research targets, the focus may be on characterisation of crop-specific microbiomes, splitting cultivar-related and environment-related microbiomes, partitioning microbiomes’ exact contributions to plant fitness, stress response and yield and analysis of metagenomes and microbiome metabolic pathways [281]. Insights gained from such investigations are key in enabling the custom design of microbiome-based techniques and methods for improving crop growth and stress tolerances [277,281]. Moreover, in view of climate change and the apparent geographical variations in extreme weather events and environmental stress consequences, metagenomics potentially assists in identifying the most beneficial crop–microbiomes combinations and effective mapping of suitable crops to specific locations [25].

## 8. Challenges and Perspectives

Developing stress-tolerant cereal crops is daunting. Despite undeniably revolutionising the dissection of molecular mechanisms and identification of key candidate traits underpinning crop plants’ adaptation to various environmental stresses [35], the use of omics techniques has its own limitations. Firstly, several of the key candidate stress-responsive genes and proteins remain largely of unknown function due to limitations in their functional characterisation [288]. Owing to limited functional annotation capabilities of some bioinformatics tools, voluminous multiomics data remain just unanalysed “big data” in databases, with little biological relevance [289].

Secondly, the heterogeneity of multiomics data (endowed with different inherent biases, noises and scaling issues) from various platforms makes its unification and normalisation difficult. Moreover, its interpretation is complicated by the intricate nature of biological networks and omics levels that demand efficient analysis tools [290]. Fortunately, advances in computational biology and data analysis approaches, coupled with machine learning (ML), could help disentangle the complex “big” data, providing the much-needed new information of biological significance, including identification of novel genes and proteins, and revealing their functions in crop stress tolerance and yield [291,292,293].

Thirdly, most of the key candidate stress-responsive genes identified are yet to be functionally verified. One of the reasons is that cereal crop species such as sorghum are not amenable or have low transformation efficiencies due to recalcitrance [294]. To address the problem of transformation incompatibilities in such cereal crops, modern gene-editing technologies, such as nanoparticle-based CRISPR-Cas delivery systems, can be used [68,295]. However, since abiotic stress tolerance is a complex multigenic trait, the use of gene editing, which often targets single or a few genes, in the development of new stress-tolerant cereal crop cultivars may be limited. Moreover, gene editing depends on the mechanistic understanding of the specific genes underpinning plant tolerance to stress; yet, these causal genes have yet to be pinpointed and validated, especially under field conditions [296]. This is further convoluted by the complex crosstalk among several gene regulatory networks and stress signalling pathways. Nonetheless, we anticipate the amalgamation of multiomics, GWAS, genomic selection, phenomics and genomic prediction approaches to ease the identification of the major genetic factors regulating stress tolerance, whilst computational biology, Synbio and ML will facilitate deconstruction and elucidation of complex stress response networks, thereby aiding stress tolerance engineering in cereal crops [291,292,297,298].

Moreover, several studies have functionally verified candidate genes and networks, mainly in the model species Arabidopsis and not in cereal crops [299]. However, cross-species validation may not fully reflect gene or protein function in cereals due to differences in plant/crop physiology and regulatory context [300]. Thus, future studies involving gene knockouts or overexpression directly in cereals would better elucidate their native functional roles. Further, most of the identified stress-responsive genes are yet to be validated under field conditions. Whereas most stress-response experiments are performed under controlled lab environments, where usually single stresses are studied, crop plants growing in the field face unstable, harsh environmental conditions, often involving complex multiple stresses [301,302]. Therefore, translation of “impressive” lab results to the field (outside lab resource-constrained) settings has not yielded expected outcomes with regard to crop stress tolerance. Going forward, carrying out stress-response studies, especially examination of the physiological responses or the causal inferences between phenotypes and candidate genes, under actual field conditions, may provide reliable results and help in translating foundational discoveries to field crops.

## 9. Conclusions and Future Outlook

Cereal crops, like any other plants, are routinely exposed to a variety of abiotic and biotic stresses, against which they must develop appropriate responses. Therefore, creating stress-tolerant crops requires first gaining a mechanistic understanding of the plant biological processes and fundamental mechanisms underlying responses to those stresses. Here, we have discussed how the recent progress in genomics technologies and approaches, including third-generation sequencing platforms, long-read sequences, pangenomes, GWAS and multi-omics, has considerably aided the exploration of mechanisms underlying crops’ tolerance to abiotic and biotic stresses. Some of the identified candidate genes and metabolic pathways have been deployed in genomics-assisted or marker-assisted breeding programs via molecular breeding approaches or genetic-engineering methodologies. Integrating other new and innovative approaches such as genome-editing techniques, HT3Ps, modern plant breeding and synthetic biology could facilitate rapid identification and introgression of novel and key stress-responsive traits into elite crops and create climate-smart cereal cultivars (Figure 2). Going forward, we anticipate single-cell omics approaches to dominate the landscape of identifying, monitoring and analysing spatiotemporal expressions of key stress-responsive genes and metabolites [29,303,304]. This will be supported by advances in HT3Ps, machine learning and artificial intelligence [292]. Resolving biological questions at the single-cell level will reveal novel insights into the spatiotemporal interplay among transcriptional, metabolic, phytohormonal and epigenetic regulatory networks, thereby aiding the design of climate-resilient cereals [305]. Moreover, synthetic biology is expected to become popular in metabolic pathway engineering for stress tolerance in crops, aided by novel genome-editing technologies [292,306]. Overall, the advances in plant functional genomics approaches, in integration with recent plant breeding, genome-editing and computational biology techniques, will simplify the untangling of complex mechanisms of plant stress tolerance and facilitate the design of stress-resilient cereals for a food-secure future.

## Data Availability

No new data was generated in this research.

## Supplementary Materials

The following supporting information can be downloaded at: https://www.mdpi.com/article/10.3390/plants14162459/s1, Supplementary Table S1. Selected comparative transcriptomic studies revealing abiotic stress-responsive genes and tolerance mechanisms in major cereal crops. Supplementary Table S2. Selected comparative proteomics studies revealing abiotic stress-responsive proteins and tolerance mechanisms in major cereal crops. Refs. [307,308,309,310,311,312,313,314,315,316,317,318,319,320,321,322,323,324,325,326,327,328,329,330,331,332] are cited in the Supplementary Materials.
