# Supplementary material for: Advances in Functional Genomics for Exploring Abiotic Stress Tolerance Mechanisms in Cereals
Source: Plants (Basel). 2025 Aug 8;14(16):2459. doi: 10.3390/plants14162459 (PMC12389445; doi:10.3390/plants14162459)
Supplement: Supplementary file 1 [file plants-14-02459-s001.zip › Supplementary Table S1- Selected Transcriptomics Studies.pdf]

plants

MDPI

Supplementary Table S1. Selected comparative transcriptomic studies revealing abiotic stress-responsive genes and tolerance mechanisms in major cereal crops.

Abiotic stress	Crop species	Genotypes	Context	Outcome/ Key findings	Reference
Drought	Wheat (*Triticum aestivum*)	Drought-tolerant T13 and drought-susceptible T2	Integrated transcriptome and metabolome analyses of differing wheat genotypes under drought at seedling stage.	Flavonoids and phenolic acids metabolisms were related with wheat seedling's drought tolerance, with their biosynthesis-related DEGs or DEMs possibly underlining the difference in drought tolerance.	[1]
Drought	Foxtail millet (*Setaria italica*)	Drought-tolerant Jigu39, moderately-tolerant Jingu21, and drought-sensitive Longgu16	Comparative transcriptome analysis of 3 differing foxtail millet genotypes under drought.	2954, 1531, and 2344 DEGs were identified in the three genotypes, respectively. Key drought-responsive DEGs were significantly enriched in photosynthesis, chlorophyll metabolism, and carbohydrate metabolism pathways, with 32 novel foxtail millet drought-responsive candidate genes identified.	[2]
Drought and salinity	Wheat	Drought/salt tolerant JM22 and salt sensitive YM20	Genome-wide transcriptome analysis of wheat cultivars response to drought (10% soil moisture) and salinity (100 mM NaCl) stresses.	10 DEGs, annotated to cellular process, metabolic process, osmotic regulation, and MAPK signaling pathway, were co-identified as drought and salinity tolerance associated DEGs contributing to better stress tolerance in JM22.	[3]
Drought	Maize	Drought-tolerant CML69 and susceptible LX9801 inbred lines	Comparative transcriptomic and physiological analyses of maize leaf tissues of seedlings subjected to 3 to 5 days of drought treatment.	Among other key results, the tolerant line showed significantly higher leaf RWC, and lower electrolyte leakage and MDA levels than the susceptible line, whch possibly contributed to its better drought tolerance.	[4]
Waterlogging	Maize	Tropical line Suwan-2 and temperate line Cim-3	Comparative physiological and transcriptomic analysis of tropical and temperate maize inbred line tolerance to waterlogging stress.	Crucial waterlogging-responsive DEGs in Suwan-2 were related to TF modulation, cellular redox homeostasis maintenance, and plant hormone biosynthesis regulation.	[5]
Drought	Rice	Drought-tolerant Nagina 22 and drought-sensitive IR 64	Comparative analysis of immature rice panicles from plants grown under control and drought treatments at the reproductive stage.	Majority of drought-responsive DEGs in the tolerant genotype Nagina22 were associated with phytohormone, redox signaling, and secondary metabolite biosynthesis.	[6]
Drought	Maize	Drought-tolerant SD609 and drought-sensitive SD902	Transcriptomic and physiological analysis of maize responses to drought stress.	Higher drought tolerance of SD609 than SD902 was attributed to its robust stress defense capabilities, IAA signal transduction, and more stable photosynthesis.	[7]
Salinity	Wheat	Salt tolerant Neixiang188 and salt-sensitive Barra	RNA-Seq analysis of wheat root tissues at 6 h and 24 h after salinity treatment.	Neixiang188 exhibited better salinity tolerance than Barra owing to its superior ion homeostasis, ROS detoxification, and osmotic adjustment abilities.	[8]

Salinity	Wheat	Salt-tolerant Cangmai6005 (CM6005) and salt-sensitive Kenong9204 (KN9204)	Phenotypic screening and transcriptomic analysis of different wheat genotypes under salinity stress treatments (tests).	More salt-tolerance related DEGs were up-regulated in CM6005 than in KN9204, and among them were those associated with trehalose-related processes, MAPK signal transduction, jasmonic acid-related pathways, etc.	[9]
Salinity	Wheat	Saudi cultivar Najran	Comparative RNA-seq analysis of roots and shoots of bread wheat plants grown under unstressed control condition (0 mM NaCl) and salt treatment (200 mM NaCl).	Most significantly regulated salt tolerance-related pathways in the root and shoot tissues were glutathione metabolism and biosynthesis of secondary metabolites (eg., phenylpropanoids and galactose metabolism).	[10]
Salinity	Rice	Salt-tolerant HH11 and salt-sensitive IR29 cultivars	Transcriptome analysis of rice genotypes treated with 200 mM NaCl solution for 0 h, 6 h, 24 h and 48 h at the three leaf stage.	HH11 showed more favorable antioxidant and osmotic adjustments than IR29 upon salt strees exposure, thus, better salt tolerance.	[11]
Heat	Rice (indica and japonica)	Two rice varieties, 93–11 (indica) and ZH11 (japonica)	Analysis of transcriptomic changes in the root tissues of two rice varieties under heat stress and during recovery.	The two varieties exhibited several shared, and uniquely enriched pathwaysa, reflecting distinct responses to heat stress. WGCNA revealed *OsMAPK3* as a novel hub gene for rice HS response.	[12]
Heat stress (HS)	Wheat	Heat tolerant genotype WH-730	Transcriptomic analysis of a heat tolerant wheat genotype response to control and heat treatment conditions.	5610 heat-responsive DEGs were identified, and participate in HS response pathways, eg., HSPs, antioxidant defense, and metabolic adjustments. Among them, peroxidase was dorminant, enhancing HS tolerance, possibly via regulation of lignin biosynthesis.	[13]
Heat	Rice	Heat-tolerant T11 and heat-sensitive T15	Transcriptome analysis of wheat response to HS (42 °C) at different exposure durations (0 min to 10h).	Tolerant cultivar T11 responded more rapidly and intensively to HS than the sensitive cultivar, with MAPK signaling pathway identiifed as the specific early-response pathway of T11.	[14]
Heat	Wheat	HS tolerant Raj3765	Transcriptome analysis of wheat HS (42 °C for 6h) response.	7990 DEGs were identified, with key HS-responsive DEGs enriched mainly in the metabolic and secondary metabolites biosynthesis pathways. MYB, bHLH, WRKY, NAC, and ERF TFs were abundant and suggested to underpin HS tolerance.	[15]
Drought (DS) and cold stress (CS)	Wheat	Stress-resistant Saratovskaya 29 (S29) and stress-sensitive Yanetzkis Probat (YP) cultivars.	Comparative transcriptome analysis of differing wheat genotypes` responses to drought and cold stresses.	Key stress-responsive DEGs were those related to photosynthetic apparatus, WRKY TFs, and zinc transporters. Expression of photosynthetic apparatus-related DEGs decreased	[16]

				in both cultivars under DS, and expression of chloroplast and thylakoid membranes decreased under CS.	
Cold	Rice	Cold-tolerant cultivar Huaidao5 and Cold-sensitive cultivar Huaidao9	Differential expression and co-expression network analyses of rice panicle and flag leaf transcriptomes under reproductive-stage cold stress.	Huaidao5 showed better panicle tolerance to cold stress due to higher expression levels of cold-responsive genes in related pathways, eg. MAPK signaling pathway, glutathione metabolism, plant hormone signal transduction, etc.	[17]
Cold and anaerobic	Rice	Tolerant genotype Darij and susceptible line 4610	Comparative global gene expression analysis of two contrasting rice genotypes under cold, anaerobic, and combined stresses during germination.	5571 DEGs, 7206 DEGs, and 13279 DEGs were identified under anaerobic, cold, and combined stress, respectively. DEGs related to CHO metabolic process, glucosyltransferase activity, etc., were enriched across all stresses.	[18]
Chilling	Rice	Tolerant Koshihikari (Kos) and sensitive Kasalath (Kas)	Comparative transcriptome and morphophysiological analyses of rice cultivars tolerance to chilling (17 °C) stress.	Kos exhibited better chilling stress tolerance than Kas due to its enrichment of carotenoid biosynthesis pathway (CBP) and higher expression of CBP-related DEGs at 17 °C.	[19]
Cadmium	Rice	Cd resistant T207 and Cd sensitive S276	Comparative physiological and transcriptomic analyses of wheat genotypes response to cadmium stress.	Cd toxicity affected the transcriptional profiles of genes involved in AsA-GSH cycle, and a larger % of these DEGs showed higher expression in T207 than S276.	[20]
Cadmium	Rice	ZZ143 (low grain Cd) and YX409 (high grain Cd)	Genotypes subjected to 100μmol/L Cd stress for 10 days.	ZZ143 showed higher root Cd tolerance than the susceptible genotype, possibly due to its greater root sulphur assimilation, and higher number of Cd-responsive DEGs and pathways, eg., secondary metabolites biosynthesis, MAPK signaling, etc	[21]
Aluminium	Wheat	Fielder (Al-tolerant and P-efficient) and Ardito (Al-sensitive and P-inefficient)	Genotypes exposed to 50 μM Al and 2 μM Pi (LP) in hydroponic solutions for 4days.	Aluminium tolerance related DEGs were significantly enriched in the phenylpropanoid, flavonoid biosynthesis, and carbohydrate metabolism pathways in the AL-tolerant genotype.	[22]
Low nitrogen (N)	Barley	Low N tolerant line A9-29 and low N sensitive variety Hua 30	Comparative transcriptome analysis of barley root responses to low N stress.	A9-29 specific low N stress-responsive DEGs were mainly enriched in energy metabolism, lipid metabolism, and amino acids metabolism. Among key genes, ERFs TFs and IAA-related genes were specific to A9-29.	[23]
Low nitrogen	Maize	Low-N-tolerant XY335 and low-N-sensitive HN138	Analysis of the maize leaf transcriptome and proteome of plants grown under low N stress at the grain filling stage.	761 DEGs were specific to XY335 and 259 DEGs specific to HN138, whilst 59 DEGs were shared under low-N-stress conditions. XY335 could better tolerate low-N stress than	[24]

				HN138, due to its robust low-N-stress sensing and signaling and increased photosynthesis efficiency.	
Low nitrogen	Barley	Nitrogen-efficient genotype W26 and nitrogen-sensitive genotype W20	Transcriptome and metabolome analyses of barley reponse to low N treatment for 3 days and 18 days, and N resupply from 18 to 21 days.	Responses of W26 and W20 to low N stress were significantly different at both transcript and metabolome levels, but glutathione (GSH) metabolism pathway was of significance in both leaf and root responses to low N stress in both genotypes.	[25]
Low nitrogen	Foxtail millet	Highly sensitive genotype Maotigu and highly tolerant genotype Dahuanggu	Transcriptome analysis of foxtail millet root tissues of seedlings grown under LN (0.1 mmol/L) and control (5 mmol/L) conditions under hydroponic conditions.	823 DEGs and 2427 DEGs were identified in Dahuanggu and Maotigu roots under low N treatment, respectively. Pathways related to plant–pathogen interaction, galactose metabolism, and flavonol biosynthesis were suggested to mediate low N tolerance in foxtail millet.	[26]
Low nitrogen	Sorghum	N-efficient (398B) and the N-inefficient (CS3541) inbred lines	Comparative phenotypic and transcriptome analysis of sorghum genotypes under low N hydroponic and field conditions	398B exhibited superior low N tolerance than CS3541 under both field and hydroponic conditions, due to its higher photosynthetic performance and sustenance of N metabolism-related enzymes activities.	[27]

Note: DEGs, differentially expressed genes; DEMs, differentially expressed metabolites; HSPs, heat shock proteins; CHO, carbohydrates; Cd, cadmium. 2

References

1. Guo, X.; Lv, L.; Zhao, A.; Zhao, W.; Liu, Y.; Li, Z.; Li, H.; Chen, X. Integrated Transcriptome and Metabolome Analysis Revealed Differential Drought Stress Response Mechanisms of Wheat Seedlings with Varying Drought Tolerance. *BMC Plant Biol.* **2025**, *25*, 571.
2. Guo, Y.; Hao, D.; Wang, X.; Wang, H.; Wu, Z.; Yang, P.; Zhang, B. Comparative Transcriptomics Reveals Key Genes Contributing to the Differences in Drought Tolerance among Three Cultivars of Foxtail Millet (Setaria Italica). *Plant Growth Regul.* **2023**, *99*, 45–64.
3. Dugasa, M.T.; Feng, X.; Wang, N.-H.; Wang, J.; Wu, F. Comparative Transcriptome and Tolerance Mechanism Analysis in the Two Contrasting Wheat (Triticum Aestivum L.) Cultivars in Response to Drought and Salinity Stresses. *Plant Growth Regul.* **2021**, *94*, 101–114.
4. Waititu, J.K.; Zhang, X.; Chen, T.; Zhang, C.; Zhao, Y.; Wang, H. Transcriptome Analysis of Tolerant and Susceptible Maize Genotypes Reveals Novel Insights about the Molecular Mechanisms Underlying Drought Responses in Leaves. *Int. J. Mol. Sci.* **2021**, *22*, 6980.
5. Yao, Q. Crucial Waterlogging-Responsive Genes and Pathways Revealed by Comparative Physiology and Transcriptome in Tropical and Temperate Maize (Zea Mays L.) Inbred Lines. *J. Plant Biol.* **2021**, *64*, 313–325.
6. Kaur, S.; Seem, K.; Duhan, N.; Kumar, S.; Kaundal, R.; Mohapatra, T. Transcriptome and Physio-Biochemical Profiling Reveals Differential Responses of Rice Cultivars at Reproductive-Stage Drought Stress. *Int. J. Mol. Sci.* **2023**, *24*, 1002.
7. Wang, Y.; Guo, H.; Wu, X.; Wang, J.; Li, H.; Zhang, R. Transcriptomic and Physiological Responses of Contrasting Maize Genotypes to Drought Stress. *Front. Plant Sci.* **2022**, *13*, 928897.
8. Wu, G.; Sun, X.; Sun, Q.; Kang, X.; Wang, J.; He, X.; Liu, W.; Xu, D.; Dai, X.; Ma, W. Genetic Variation in Wheat Root Transcriptome Responses to Salinity: A Comparative Study of Tolerant and Sensitive Genotypes. *Int. J. Mol. Sci.* **2025**, *26*, 331.
9. Wang, W.; Zou, J.; Zhang, Y.; Niu, L.; Yu, L.; Wang, Z.; Wang, F.; Zhang, S.; Yang, X. Salinity Stress Phenotyping of Wheat Germplasm under Multiple Growth Conditions and Transcriptomic Analysis of Two Wheat Varieties Contrasting in Their Salinity Stress Tolerance. *Plant Growth Regul.* **2025**, *105*, 739–757.
10. Alyahya, N.; Taybi, T. Comparative Transcriptomic Profiling Reveals Differentially Expressed Genes and Important Related Metabolic Pathways in Shoots and Roots of a Saudi Wheat Cultivar (Najran) under Salinity Stress. *Front. Plant Sci.* **2023**, *14*, 1225541.
11. Fang, X.; Mo, J.; Zhou, H.; Shen, X.; Xie, Y.; Xu, J.; Yang, S. Comparative Transcriptome Analysis of Gene Responses of Salt-Tolerant and Salt-Sensitive Rice Cultivars to Salt Stress. *Sci. Rep.* **2023**, *13*, 19065.
12. Deng, Y.; Zhu, Z.; Chen, J.; Kuang, L.; Yan, T.; Wu, D.; Gao, F. Comparative Transcriptomics of Indica and Japonica Rice Roots under Heat Stress Reveals the Crucial Role of OsMAPK3 in Heat Response. *Plant Physiol. Biochem.* **2025**, *221*, 109668.
13. Lamba, K.; Kumar, M.; Singh, V.; Chaudhary, L.; Gupta, V. Transcriptome Analysis for Heat Stress Related Genes in Wheat Genotype WH-730. *Cereal Res. Commun.* **2025**, *53*, 793–805.

14. Cai, H.; Wang, H.; Zhou, L.; Li, B.; Zhang, S.; He, Y.; Guo, Y.; You, A.; Jiao, C.; Xu, Y. Time-Series Transcriptomic Analysis of Contrasting Rice Materials under Heat Stress Reveals a Faster Response in the Tolerant Cultivar. *Int. J. Mol. Sci.* **2023**, *24*, 9408.
15. Azameti, M.K.; Ranjan, A.; Singh, P.; Gaikwad, K.; Singh, A.K.; Dalal, M.; Arora, A.; Rai, V.; Padaria, J.C. Transcriptome Profiling Reveals the Genes and Pathways Involved in Thermo-Tolerance in Wheat (Triticum Aestivum L.) Genotype Raj 3765. *Sci. Rep.* **2022**, *12*, 14831.
16. Konstantinov, D.K.; Zubairova, U.S.; Ermakov, A.A.; Doroshkov, A.V. Comparative Transcriptome Profiling of a Resistant vs Susceptible Bread Wheat (Triticum Aestivum L.) Cultivar in Response to Water Deficit and Cold Stress. *PeerJ* **2021**, *9*, e11428.
17. Niu, Y.; Fan, S.; Cheng, B.; Li, H.; Wu, J.; Zhao, H.; Huang, Z.; Yan, F.; Qi, B.; Zhang, L. Comparative Transcriptomics and Co-Expression Networks Reveal Cultivar-Specific Molecular Signatures Associated with Reproductive-Stage Cold Stress in Rice. *Plant Cell Rep.* **2023**, *42*, 707–722.
18. Thapa, R.; Tabien, R.E.; Johnson, C.D.; Septiningsih, E.M. Comparative Transcriptomic Analysis of Germinating Rice Seedlings to Individual and Combined Anaerobic and Cold Stress. *BMC Genomics* **2023**, *24*, 185.
19. Zhang, P.; Wu, X.; Chen, Y.; Ji, G.; Ma, X.; Zhang, Y.; Xiang, J.; Wang, Y.; Wang, Z.; Li, L. Comparative Transcriptome Combined with Morphophysiological Analyses Revealed Carotenoid Biosynthesis for Differential Chilling Tolerance in Two Contrasting Rice (Oryza Sativa L.) Genotypes. *Rice* **2023**, *16*, 52.
20. Zhang, T.; Xiao, J.; Zhao, Y.; Zhang, Y.; Jie, Y.; Shen, D.; Yue, C.; Huang, J.; Hua, Y.; Zhou, T. Comparative Physiological and Transcriptomic Analyses Reveal Ascorbate and Glutathione Coregulation of Cadmium Toxicity Resistance in Wheat Genotypes. *BMC Plant Biol.* **2021**, *21*, 1–15.
21. Zhao, S.; Zhang, Q.; Xiao, W.; Chen, D.; Hu, J.; Gao, N.; Huang, M.; Ye, X. Comparative Transcriptomic Analysis Reveals the Important Process in Two Rice Cultivars with Differences in Cadmium Accumulation. *Ecotoxicol. Environ. Saf.* **2023**, *252*, 114629.
22. Luo, D.; Li, Q.; Pang, F.; Zhang, W.; Li, Y.; Xing, Y.; Dong, D. Commonalities and Specificities in Wheat (Triticum Aestivum L.) Responses to Aluminum Toxicity and Low Phosphorus Revealed by Transcriptomics and Targeted Metabolomics. *Int. J. Mol. Sci.* **2024**, *25*, 9273.
23. Gao, R.; Zhou, L.; Guo, G.; Li, Y.; Chen, Z.; Lu, R.; Liu, C.; Chen, J. Comparative Analysis of Root Transcriptome of High-NUE Mutant and Wild-Type Barley under Low-Nitrogen Conditions. *Agronomy* **2023**, *13*, 806.
24. Li, J.; Zenda, T.; Liu, S.; Dong, A.; Wang, Y.; Liu, X.; Wang, N.; Duan, H. Integrated Transcriptomic and Proteomic Analyses of Low-Nitrogen-Stress Tolerance and Function Analysis of ZmGST42 Gene in Maize. *Antioxidants* **2023**, *12*, 1831.
25. Wang, G.; Wang, J.; Yao, L.; Li, B.; Ma, X.; Si, E.; Yang, K.; Li, C.; Shang, X.; Meng, Y. Transcriptome and Metabolome Reveal the Molecular Mechanism of Barley Genotypes Underlying the Response to Low Nitrogen and Resupply. *Int. J. Mol. Sci.* **2023**, *24*, 4706.

26. Wu, J.; Chen, L.; Yang, Z.; Lu, J.; Yang, J.; Li, N.; Shi, H. Transcriptomics Uncovers Pathways Mediating Low-Nitrogen Stress Tolerance in Two Foxtail Millet Varieties. *Agriculture* **2025**, *15*, 628.
27. Liu, C.; Gu, W.; Liu, C.; Shi, X.; Li, B.; Zhou, Y. Comparative Phenotypic and Transcriptomic Analysis Reveals Genotypic Differences in Nitrogen Use Efficiency in Sorghum. *Plant Physiol. Biochem.* **2024**, *215*, 109028.
