# Supplementary material for: Advances in Functional Genomics for Exploring Abiotic Stress Tolerance Mechanisms in Cereals
Source: Plants (Basel). 2025 Aug 8;14(16):2459. doi: 10.3390/plants14162459 (PMC12389445; doi:10.3390/plants14162459)
Supplement: Supplementary file 1 [file plants-14-02459-s001.zip › Supplementary Table S2 - Proteomics studies for abiotic stress.pdf]

plants

Supplementary Table S2. Selected comparative proteomics studies revealing abiotic stress-responsive proteins and tolerance mechanisms in major cereal crops.

Abiotic stress	Crop species	Genotypes	Context	Outcome/ Key findings	Reference
**Proteomics**					
Drought	Wheat	Drought-tolerant BW35695 and drought-sensitive BW4074	Physiological, biochemical, and iTRAQ leaf proteome analyses of wheat responses to drought.	Tolerant variety showed greater osmotic adjustment , antioxidant capacity, and high upregulation of protein synthesis-related proteins, contributing to better stress tolerance.	[1]
Drought	Maize	Drought-tolerant YE8112 and drought-sensitive MO17	Physiological and iTRAQ leaf proteome analyses of maize responses to drought.	A total of 721 DAPs were identified. Most significantly enriched DAPs in YE8112 were associated with the photosynthesis antenna proteins pathway, and YE8112 had better tolerance due to its activation of photosynthesis proteins involved in balancing light capture and utilization.	[2]
Heat	Rice	Heat-olerant variety 9311 and sensitive variety Guangluai4 (GLA4)	Phosphoproteomic analysis of high temp (30–38 °C for 1 to 9 days)-induced changes in indica rice developing grains.	A total of 9994 phosphosites from 3216 phosphoproteins were identified in all endosperm samples. Several HS-induced consensus phosphorylation motifs were identified, and revealed a core set of HS-responsive protein kinases, splicing factors, and regulatory factors, especially those involved in starch metabolism.	[3]
DS and elevated temp (ET)	Barley	7 spring barley RILs (hybrids of European and Syrian accessions)	LC-MS based proteomic analysis of barley flag leaf response to drought and ET (20/30 °C night/day).	several protein accumulation changes under DS, ET and combined stresses were identified, including for photosynthetic apparatus-related proteins. Dehydrins were found among universally stress-responsive proteins.	[4]
Waterlogging	Wheat	Tolerant XM 55 and sensitive genotypes YM 158	iTRAQ proteomic analysis of wheat responses to waterlogging stress.	Of the 7710 DAPs identified, 16 were distinct between the 2 cultivars under stress; 11 DAPs were up-regulated and 5 down-regulated. 9 DAPs, including DEAD-box ATP-dependent RNA helicase 3, responded to waterlogging with non-cultivar specificity.	[5]
Salinity	Pearl millet (*Pennisetum glaucum*)	Tolerant (Tol) and sensitive (Sen) accessions	2DE based whole proteome analysis analysis of pearl millet reponse to 150 mm NaCl treatment	295 and 315 protein spots were identified in tolerant and sensitive accession, respectively. salinity tolerance of the tolerant accession was attributed to its higher upregulation stress-responsive proteins.	[6]
Salinity	Wheat	Kharchia-65 (highly salt-tolerant) and	LC–MS/MS based proteomic analysis of wheat responses to 0 and 300 mM NaCl treatment for 48 h.	21863 proteins and 5133 protein groups were identified. There was higher up-regulation of stress-responsive proteins, eg., auxin-responsive, peroxidase, etc., in tolerant genotype and comparative down-regulation in susceptible genotype.	[7]

		PBW-373 (salt-sensitive)			
Low temperature (LT)	Maize	LT tolerant Gurez local and LT-sensitive GM6	2D-PAGE based proteomic analyis of maize leaf responses to low temp (6 °C) exposure for 12h at 3 leaf stage	19 and 10 proteins were identified in Gurez local and GM6, respectively, including 3 novel abiotic stress- and LT-responsive proteins (eg., nodulin-like protein) identified from Gurez local.	[8]
Aluminium (Al)	Barley	Al-sensitive barley cultivar ZU9	TMT-based quantitative proteomic analysis of barley response to aluminium stress under phosphorus-*Piriformospora indica* interaction	DEPs were mostly enriched in the phenylpropanoid biosynthesis pathway, among which peroxidases were prominent. *P. indica* in combination with P helped barley plants to endure Al-induced stress by modulating antioxidative defense system.	[9]
Low inorganic phosphorus (Pi) availability	Wheat	Higher PUE genotype TM98 and a lower PUE genotype H4399	Label-free quantitative proteomic analysis of wheat leaf responses to low Pi avaiability	2110 high-confidence proteins were identified, among them 244 and 133 DAPs under Pi deficiency in H4399 and TM98, respectively. Abundance of energy metabolism-related protein was decreased by Pi deficiency in H4399 shoots, but not in PUE-efficient genotype TM98.	[10]
Drought	Sorghum	Drought-sensitive S4 and S4-1, and drought-resistant T33 and T14	nano-LC-MS/MS based leaf proteome analysis of sorghum response to drought.	A total of 3927 proteins were quantitated, with 46, 36, 35, and 102 DAPs identified in S4, S4-1, T14, and T33 varieties, respectively. Tolerant genotypes showed enhanced TCA cycle, sphingolipid biosynthesis, and influenced aminoacyl-tRNA biosynthesis.	[11]
Heat	Rice	Tolerant Huang Huazhan (HZ) and susceptible Yang Dao6 (YD) varities	Physiological and proteomic analysis of rice anthers responses to heat stress (38°C, 6 hours for 3 days).	Antioxidant and osmoregulatory capacities, as well as GA/ABA ratio were higher in HZ than YD. HS-responsive DAPs, mainly involved in phenylpropanoid biosynthesis, ubiquitin-mediated proteolysis and CHO metabolism pathways, were highly upregulated in HZ than YD.	[12]
Heat	Rice	Moroberekan (Japonica, heat sensitive), IR64 (Indica, moderately heat tolerant), and Nagina22 (Aus, heat tolerant)	Shotgun proteomics analysis of rice anther responses to short-term (ST_HS; one cycle of 42°C, 4 hours before anthesis) and long-term (LT_HS; 6 cycles of 38°C, 6 hours before anthesis) heat stress.	The heat response in Nagina22 was associated with its capacity for adequate metabolic control and cellular homeostasis, which may be critical for its higher reproductive thermotolerance.	[13]
Drought	Maize	Drought-tolerant Chang 7-2 and	iTRAQ based proteomic analysis of maize seedling root responses to 20%	7723 DEPs were identified, 1243 of which were significantly differentially expressed in Chang 7-2 following drought stress. Higher drought tolerance of Chang 7-2 root system	[14]

		drought-sensitive TS141 varieties	polyethylene glycol 6000 (PEG 6000)-simulated drought stress.	was attributed to a stronger water retention capacity, antioxidant enzyme activities, osmotic stabilization of plasma membrane proteins; and improved lignification, among other mechanisms.	
Salinity	Pearl millet	Salt-tolerant IC 325825 and salt-sensitive IP 17224 genotypes	Proteomic analysis of pearl millet responses to 150 mM NaCl treatment.	The salt-tolerance potential of IC325825 was associated with its ability to maintain intracellular osmotic, ionic, and redox homeostasis and membrane integrity under stress.	[15]
Drought	Maize	Drought-tolerant SD609 and drought-sensitive SD902 cultivars	Physiological and proteomic analyses of maize responses to drought.	198 and 102 DEPs were identified in SD609 and SD902, respectively. tolerant genotype SD609 showed upregulated expression of proteins associated with photosynthesis, antioxidants enzymes and molecular chaperones, which possibly contributed to its better tolerance.	[16]
Drought	Maize	Maize inbred line B73	iTRAQ based proteomic analysis of maize leaf responses to drought.	3063 DAPs were identified, among which the abundance of 214 and 148 proteins increased and decreased, respectively, after 3 day drought treatment. DAPs were mainly involved in cell redox homeostasis, cell wall organization, photosynthesis, ABA biosynthesis, and stress-response processes.	[17]
Phophorus stavation	Rice	High-yielding rice cultivar Pusa-44 and its near-isogenic line (NIL)-23	LFQ-based proteomic analysis of rice shoot and root responses to P-stavation stress.	681 and 567 DEPs were identified in shoot of Pusa-44 and NIL-23, respectively. Similarly, 66 and 93 DEPs were identified in root of Pusa-44 and NIL-23, respectively. These P-starvation responsive DEPs were mainly involved in metabolic processes like photosynthesis, starch-, sucrose-, energy-metabolism, TFs (mainly ARF, ZFP, HD-ZIP, MYB), and phytohormone signaling.	[18]
Low N	Maize	Low N-efficient hybrid XY335 and low N-inefficient HN138	Proteomic analysis of maize leaf responses to low N stress at the 1w leaf stage.	Low N tolerant hybrid responded to low N stress through lignin biosynthesis, ubiquitin-mediated proteolysis, and stress defense proteins.	[19]

Note: DEGs, differentially expressed genes; DAPs, differentially abundant proteins; DEMs, differentially expressed metabolites; HSPs, heat shock proteins; CHO, carbohydrates; Cd, cadmium; The experiment included two genotypes of wheat, KH-65 and PBW-373; LC-MS/MS, liquid chromatography combined with mass spectrometry analysis; nano-LC-MS/MS, nano-scale liquid chromatography mass spectrometry; 2D-PAGE, two dimensional gel electrophoresis; LFQ-based, label-free quantification based.

References

1. Moloi, S.J.; Alqarni, A.O.; Brown, A.P.; Goche, T.; Shargie, N.G.; Moloi, M.J.; Gokul, A.; Chivasa, S.; Ngara, R. Comparative Physiological, Biochemical, and Leaf Proteome Responses of Contrasting Wheat Varieties to Drought Stress. *Plants* **2024**, *13*, 2797.
2. Zenda, T.; Liu, S.; Wang, X.; Jin, H.; Liu, G.; Duan, H. Comparative Proteomic and Physiological Analyses of Two Divergent Maize Inbred Lines Provide More Insights into Drought-Stress Tolerance Mechanisms. *Int. J. Mol. Sci.* **2018**, *19*, 3225, doi:10.3390/ijms19103225.
3. Pang, Y.; Hu, Y.; Bao, J. Comparative Phosphoproteomic Analysis Reveals the Response of Starch Metabolism to High-Temperature Stress in Rice Endosperm. *Int. J. Mol. Sci.* **2021**, *22*, 10546.
4. Mikołajczak, K.; Kuczyńska, A.; Krajewski, P.; Kempa, M.; Witaszak, N. Global Proteome Profiling Revealed the Adaptive Reprogramming of Barley Flag Leaf to Drought and Elevated Temperature. *Cells* **2023**, *12*, 1685.
5. Yang, R.; Li, M.; Harrison, M.T.; Fahad, S.; Wei, M.; Li, X.; Yin, L.; Sha, A.; Zhou, M.; Liu, K. iTRAQ Proteomic Analysis of Wheat (Triticum Aestivum L.) Genotypes Differing in Waterlogging Tolerance. *Front. Plant Sci.* **2022**, *13*, 890083.
6. Jha, S. Proteome Responses of Pearl Millet Genotypes under Salinity. *Plant Gene* **2022**, *29*, 100347.
7. Yadav, R.; Santal, A.R.; Singh, N.P. Comparative Root Proteome Analysis of Two Contrasting Wheat Genotypes Kharchia-65 (Highly Salt-Tolerant) and PBW-373 (Salt-Sensitive) for Salinity Tolerance Using LC–MS/MS Approach. *Vegetos* **2022**, *35*, 133–139.
8. Ramazan, S.; Jan, N.; John, R. Comparative Protein Analysis of Two Maize Genotypes with Contrasting Tolerance to Low Temperature. *BMC Plant Biol.* **2023**, *23*, 183.
9. Feng, Q.; Sehar, S.; Zhou, F.; Wei, D.; Askri, S.M.H.; Ma, Z.; Adil, M.F.; Shamsi, I.H. Physiological and TMT-Based Quantitative Proteomic Responses of Barley to Aluminium Stress under Phosphorus-Piriformospora Indica Interaction. *Plant Physiol. Biochem.* **2023**, *196*, 634–646.
10. Zheng, L.; Wang, R.; Zhou, P.; Pan, Y.; Shen, R.; Lan, P. Comparative Physiological and Proteomic Response to Phosphate Deficiency between Two Wheat Genotypes Differing in Phosphorus Utilization Efficiency. *J. Proteomics* **2023**, *280*, 104894.
11. Li, Y.; Tan, B.; Wang, D.; Mu, Y.; Li, G.; Zhang, Z.; Pan, Y.; Zhu, L. Proteomic Analysis Revealed Different Molecular Mechanisms of Response to PEG Stress in Drought-Sensitive and Drought-Resistant Sorghums. *Int. J. Mol. Sci.* **2022**, *23*, 13297.
12. Guo, H.; Tao, W.; Gao, H.; Chen, L.; Zhong, X.; Tang, M.; Gao, G.; Liang, T.; Zhang, X. Physiological Traits, Gene Expression Responses, and Proteomics of Rice Varieties Varying in Heat Stress Tolerance at the Flowering Stage. *Front. Plant Sci.* **2024**, *15*, 1489331.
13. Kumar, R.; Ghatak, A.; Goyal, I.; Sarkar, N.K.; Weckwerth, W.; Grover, A.; Chaturvedi, P. Heat-Induced Proteomic Changes in Anthers of Contrasting Rice Genotypes under Variable Stress Regimes. *Front. Plant Sci.* **2023**, *13*, 1083971.

14. W, Z.; Y, P.; X, Z.; B, W.; F, C.; B, R.; Z, Z.; Q, G.; Y, D. Comparative Proteomics Analysis of the Seedling Root Response of Drought-Sensitive and Drought-Tolerant Maize Varieties to Drought Stress. *Int. J. Mol. Sci.* **2019**, *20*, doi:10.3390/ijms20112793.
15. Jha, S.; Maity, S.; Singh, J.; Chouhan, C.; Tak, N.; Ambatipudi, K. Integrated Physiological and Comparative Proteomics Analysis of Contrasting Genotypes of Pearl Millet Reveals Underlying Salt-responsive Mechanisms. *Physiol. Plant.* **2022**, *174*, e13605.
16. Li, H.; Yang, M.; Zhao, C.; Wang, Y.; Zhang, R. Physiological and Proteomic Analyses Revealed the Response Mechanisms of Two Different Drought-Resistant Maize Varieties. *BMC Plant Biol.* **2021**, *21*, 1–15.
17. Ren, W.; Shi, Z.; Zhou, M.; Zhao, B.; Li, H.; Wang, J.; Liu, Y.; Zhao, J. iTRAQ-Based Quantitative Proteomic Analysis Provides Insight into the Drought-Stress Response in Maize Seedlings. *Sci. Rep.* **2022**, *12*, 9520.
18. Prathap, V.; Kumar, S.; Tyagi, A. Comparative Proteome Analysis of Phosphorus-Responsive Genotypes Reveals the Proteins Differentially Expressed under Phosphorous Starvation Stress in Rice. *Int. J. Biol. Macromol.* **2023**, *234*, 123760.
19. Wang, Y.; Wang, N.; Liu, S.; Dong, A.; Zenda, T.; Liu, X.; Li, J.; Duan, H. Comparative Proteomic Analysis of Two Contrasting Maize Hybrids' Responses to Low Nitrogen Stress at the Twelve Leaf Stage and Function Verification of ZmTGA Gene. *Genes* **2022**, *13*, 670, doi:10.3390/genes13040670.
